# Describing medicine therapy management of type 2 diabetes mellitus at primary health care facilities in Cape Town

**DOI:** 10.4102/hsag.v24i0.1051

**Published:** 2019-10-16

**Authors:** Khathatso B. Monanabela, Mea van Huyssteen, Renier Coetzee

**Affiliations:** 1 School of Pharmacy, Faculty of Natural Sciences, University of the Western Cape, Cape Town, South Africa

**Keywords:** Glycated haemoglobin, South Africa, Rational medicine use, Therapy monitoring, Type 2 diabetes mellitus

## Abstract

**Background:**

Rational medicine use aims to optimise chronic disease management and prevent episodes of hospitalisation that economically burden the health care system. Diabetes mellitus is one of the most prevalent chronic diseases globally, yet more than 60% of patients with diabetes are not optimally managed according to their therapeutic glycaemic targets.

**Aim:**

To describe the use of glycated haemoglobin (HbA1c) and fasting plasma glucose results in guiding treatment changes in patients with type 2 diabetes mellitus.

**Setting:**

Public sector primary health care facilities in the Cape Town Metropolitan Region in South Africa.

**Method:**

Retrospective, descriptive study design was employed. Data for an 18-month period were collected during 2014 and 2015. Data were collected from patient medical records and included baseline demographics, laboratory monitoring tests and the patients’ last three prescriptions.

**Results:**

The study consisted of 575 participants (64% female) with an average age of 57 (± 11.38) years. The average baseline HbA1c for 493 participants with at least one result was 8.78% (± 1.63), and only 28% of these participants reached the glycaemic target at consequent consultations. HbA1c levels were available to guide 245 prescription changes, of which 181 of these results were outside of the target range. Of these, 15.5% had appropriate therapy adjustments, 78.4% had no change or a lateral change in their follow-up prescriptions, and 6.1% had therapy adjustments opposite to what guidelines suggest.

**Conclusion:**

Glycaemic monitoring indicated consistent suboptimal glycaemic control in more than 60% of participants. Medicine prescribing patterns did not align with the prescribed local guidelines, Society for Metabolism, Endocrinology and Diabetes of South Africa (SEMDSA). The appropriate use and interpretation of HbA1c at a clinic level should be emphasised to promote rational use of medicines that minimise acute hospitalisation episodes and optimise patients’ long-term health outcomes.

## Introduction

Rational medicine use as defined by the World Health Organization (WHO 2002:1) occurs:

when patients receive the appropriate medicines, in doses that meet their own individual requirements, for an adequate period of time, and at the lowest cost to both the patients and the community. (p. 1)

One of the primary goals of rational medicine use is to optimise health expenditure by preventing episodes of hospitalisation that further burden the health care system. In the WHO’s pursuit of the responsible use of medicines, two of the seven strategic recommendations included facilitating the implementation of evidence-based guidelines for medicine use and monitoring medicine use through measuring its real-world efficacy and safety (World Health Organization 2012).

The International Diabetes Federation (IDF) estimated the global prevalence to be 415 million in 2015 (Cho et al. 2018), with an estimate that almost 2.3 million people had diabetes in South Africa (Mutyambizi et al. 2019). Large controlled clinical trials and meta-analysis have shown that improved glycaemic control, as determined through glycated haemoglobin (HbA1c) targets, were associated with a delay in the progression of the disease and reductions in macrovascular complications (Stettler et al. 2006; United Kingdom Prospective Diabetes Study (UKPDS) Group 1998). Conversely, an increased HbA1c (of above or equal to 9.5%) is a proven risk factor for the development of microvascular complications and poor quality of life in individuals with diabetes (Zhang et al. 2000). In fact, every percentage increase in HbA1c is associated with 38% risk of a macrovascular event, 40% risk in microvascular event and 38% risk of death (Zoungas et al. 2012). As the names describe, macrovascular complications involve the large arteries (stroke, angina, cardiac failure, etc.), whereas microvascular complications include those affecting the smaller blood vessels and capillaries (neuropathies, end-stage renal disease, erectile disfunction, etc.) (Triplitt, Repas & Alvarez 2019). The diabetes management guidelines for South Africa as per the Society for Metabolism, Endocrinology and Diabetes of South Africa (SEMDSA) for public health care facilities have been drafted according to the preceding evidence.

Despite the evidence that shows the benefits of attaining glycaemic goals, the management of diabetes globally is still suboptimal (Brath et al. 2016). According to the Diabetes Control and Complications Trial/Epidemiology of Diabetes Interventions and Complications (DCCT/EDIC) Research Group (2009), there is still a large gap between evidence and practice, with the majority of patients not reaching targets. In the DCCT-EDIC/EDC analysis, 81% – 87% of patients had an HbA1c > 7.0%. Two South African studies have found that only about 30% of patients reach their glycaemic targets (Amod, Riback & Schoeman 2012b; Klisiewicz & Raal 2009). Patient follow-up and therapy adjustments are important when monitoring glycaemic control and SEMDSA (Amod et al. 2012a) and the American Diabetes Association (2014) have stated their concern over the considerable variations in the regularity of follow-up visits and therapy adjustments in the management of type 2 diabetes mellitus. The worldwide diabetes ‘epidemic’ is expected to affect developing countries more than developed ones; hence studies to determine the usefulness of HbA1c as a diagnostic tool in these populations are needed, as there is a paucity of data from these communities compared to Western countries (Zemlin et al. 2011). In 2014 an expert committee of the American Diabetes Association (ADA) recommended the use of HbA1c to diagnose diabetes. Currently, there are limited published data for South Africa that relate to the management of diabetes mellitus looking at the use and interpretation of glycaemic monitoring indicators and their use to guide therapy adjustments in patients managed in the public sector at primary and community levels of care. These levels of care are most important, because the majority of relatively healthy patients are managed at this level, and this is where the most impactful interventions for preventing non-communicable disease progression and complications could happen.

The purpose of this study was to describe the use of glycated haemoglobin and fasting plasma glucose results guiding medication adjustments as part of the chronic disease management of patients with type 2 diabetes mellitus, treated at primary health care facilities in the Cape Town Metropolitan Region in South Africa. This article will describe the type of glycaemic monitoring indicators used, the level of glucose control achieved, response to glycaemic levels in terms of therapy adjustments and whether adjustments to treatment were done in accordance with evidence-based guidelines, namely the diabetes management guidelines of the Society for Metabolism, Endocrinology and Diabetes of South Africa (Amod et al. 2012). Adherence to evidence-based guidelines ensures the rational use of medicine, which may reduce complications associated with poor glucose control and benefit the long-term health of a patient (Winocour 2002).

## Method

This study was descriptive, retrospective and quantitative in design.

### Study setting

Five community health centres (CHCs) from the Tygerberg sub-district in the Cape Town Metropole were selected based on purposive sampling which was determined primarily based on the staff complement responsible for the management of type 2 diabetes mellitus patients at the facility. Facility selection criteria included the availability of a family physician, a permanent or part-time medical officer and a clinical nurse practitioner at the facility.

Chronic disease management in the public sector requires the stratification of patients with diabetes as stable or not stable. This study used the stratification categories that are summarised in Table 1. Stable chronic patients are managed at either the community or primary health care levels of care, while decompensated patients are referred to hospitals. Community health centres (CHC) at the primary health care level of the public sector are involved with the management of both stable and ‘at risk’ patients with diabetes.

**TABLE 1:** Chronic disease stratification criteria for diabetes mellitus at community health centres in the Western Cape Province.

Criteria for stratification	Gold standard	Community-based service: Stable	Primary health care: At risk	Hospital: Decompensated	
				Acute	Specialist
Glycaemic control and complexity	HbA1c target Low risk: ≤ 6.5% Majority: ≤ 7% High risk: ≤ 7.5–8.5% FPG ≤ 7 in all risk categories	Controlled at HbA1c target of ≤ 6.5% ≤ 7% ≤ 7.5–8.5% and poor short term prognosis	HbA1c above target HbA1c ˃ 11% On anti-diabetic medications And with significant co-morbidities	Diabetic ketoacidosis	Poor glycaemic control despite optimal PHC management. Pregnancy Age < 30 years
Blood pressure control and complexity	Low risk diabetic patient BP < 140/80 mmHg For a high risk BP < 150/90	Controlled at target 140/80 mmHg and 150/90 mmHg	BP ˃ 180/110 mmHg and patient is on several blood pressure medications	BP ˃ 180/110 mmHg With one of this symptoms: Headache, difficulty in breathing, visual symptoms, chest pain, leg swelling and confusion	Intolerance to multiple antihypertensives. Suspect secondary course Poor BP control despite optimal management
Total cholesterol	< 4.5 mmol/L	< 4.5 mmol/L	> 6.5 mmol/L on statins		> 7.5 mmol/L or triglycerides > 15 mmol/L despite statins

*Source*: Adapted from SEMDSA: Amod, A., Ascott-Evans, B., Berg, G., Blom, D., Brown, S., Carrihill, M. et al., 2012a, ‘The 2012 SEMDSA Guidelines for the management of type 2 diabetes’, *Journal of Endocrinology Metabolism and Diabetes of South Africa* 17(2) S1–S95. https://doi.org/10.1080/22201009.2012.10872277; and Western Cape Government Health, 2012, *Chronic disease stratification pilot report and situational analysis*, Research report dissemination (unpublished)

BP, blood pressure; FPG, fasting plasma glucose.

Patients classified as ‘at risk’ visit the CHC monthly and are monitored by the clinical nurse practitioner or medical officer at every visit. Patients classified as stable receive a 6-month repeat prescription from either the clinical nurse practitioner or medical officer at the CHC. The prescription is dispensed for 1 month and is then sent to the chronic dispensing unit where chronic medicines are issued on a 2-month basis. Patients collect their medication at a preferred pick-up point; either the CHC or a community location, such as a community hall. A register for all stable chronic patients is kept at the CHC for scheduling of appointments, and this list is also sent to the chronic dispensing unit.

After 6 months of treatment, patients are reviewed by the clinical nurse practitioner or medical officer at the CHC. If the patient’s glycaemic monitoring parameters are within target, a repeat prescription for the next 6 months is issued without a change in therapy. If the patient’s glycaemic monitoring test result is outside target, some intervention is required which could include an adjustment of therapy and monthly follow-ups; thus, only 1 month’s medication is prescribed. Table 2 reflects the stepwise treatment approach of diabetes mellitus patients according to the Department of Health: Primary Health Care Guidelines (2014).

**TABLE 2:** Stepwise pharmacotherapy approach to type 2 diabetes mellitus as per the Department of Health: Primary Health Care Guidelines (2014).

Step	Approach
Step 1 at diagnosis	Lifestyle modification
	Metformin
Step 2 if HbA1c ˃ 7% after three months or above individualised target	Metformin
	Sulphonylurea
Step 3 if HbA1c ˃ 7% after three months or remains above individualised target	Metformin plus sulphonylurea
	Basal insulin

*Source*: Department of Health, 2014, *Standard treatment guidelines and essential medicines list for South Africa: Primary health care level* (5th edn.), National Department of Health, Pretoria

### Patient selection

Non-probability sampling methods were employed, specifically convenience sampling. A complete list of all chronic patients (hypertension, asthma, diabetes, etc.) was obtained from the register for chronic patients. No electronic system was available in the participating facilities. Patient folders were screened by the researchers to identify type 2 diabetes mellitus patients. Patients with type 2 diabetes mellitus were identified based on prescriptions dispensed, which included oral anti-diabetic medication and in some cases, insulin. Patients were known neither to the researchers nor the administrative staff at the health facilities. A minimum of 100 patients per facility were reviewed for inclusion into the study. The sample was regarded as representable of the population in these communities. A sample size of 384 was calculated to be acceptable (*n* = [0.5(1–0.5)1.96^2^]/0.05^2^ = 384.16).

The target population for this study included any patient with type 2 diabetes mellitus, older than 18 years, and on anti-diabetic medication for a minimum of 6 months yet categorised as ‘stable’. The classification as ‘stable’ implied patients who were controlled at their individualised HbA1c and blood pressure levels as per the stratification criteria in Table 1. Exclusion criteria included all type 1 diabetes mellitus patients, patients younger than 18 years, and those on treatment less than 6 months, as well as those patients categorised as ‘at risk’ or ‘decompensated’.

The University of the Western Cape Research Ethics Committee (registration no. 14/9/50) and Western Cape Department of Health granted approval (reference no. 2014RP137) for the study to be conducted. The researchers were not employed or affiliated with any of the health facilities included in the study.

### Data collection and analysis

Patient medical records in the facilities were used as primary data sources. The researchers developed a data collection tool to collect information over an 18-month period to cater for three consecutive prescriptions to link treatment adjustments to corresponding HbA1c results and subsequent HbA1c tests. Input was obtained from health care practitioners, namely pharmacists, nurses and family physicians, on appropriateness of various parameters included for analysis. The data collection tool was piloted at one facility not included in the study. Amendments were made to improve ease of data collection. Data collected during the pilot phase adequately addressed the objectives of the study.

Data were collected for 2014 and 2015. Information collected included patient demographics, medical history, laboratory and clinical monitoring information, such as anti-diabetic medication regimens and glycaemic monitoring indicators, and other clinical information such as comorbidities and complications. Data from the five CHCs were combined and treated as one data set for the purposes of the analysis using Microsoft Excel^®^. SPSS version 23 was used for statistical analysis. Descriptive statistics were employed to analyse and present results. The mean and standard deviation (SD) of age, plasma glucose values, and HbA1c values were calculated.

The baseline HbA1c levels of participants were categorised into age group and risk category to establish the proportion of participants with results within and outside target ranges as per the Western Cape Province guidelines (Western Cape Government Health 2012), which are based on SEMDSA guidelines (Amod et al. 2012a). In addition to baseline measurements, this study followed the glycaemic monitoring results and the follow-up prescriptions for each participant over an 18 month period. The anti-diabetic medications were grouped according to the following regimens: mono therapy, dual therapy, mono and insulin, and dual and insulin. Mono therapy encompassed either metformin or a sulfonylurea. Dual therapy was defined as a combination of metformin and a sulfonylurea. Then ‘mono and insulin’ included either metformin and insulin or a sulfonylurea and insulin, while ‘dual and insulin’ entailed metformin, a sulfonylurea and insulin.

Prescriptions were further analysed for any changes or adjustments made in follow-up prescriptions, that is, differences between prescription one and prescription two, and between prescription two and prescription three of the same participant. Therapy adjustments were classified according to dosage changes and regimen changes. Dosage changes were classified as an increase or decrease in daily dosage. Regimen changes were classified into step-up, step-down or a lateral change. Step-up regimen changes were classified as an upward change, that is, from mono to dual therapy, from dual to dual and insulin, or from dual to mono and insulin. Step-down regimen changes were defined as the opposite of step-up. Lateral changes were a switch in drug within the same regimen, for example if a sulphonylurea was changed to metformin because of tolerability or vice versa. Finally, the appropriateness of these therapy adjustments was evaluated.

## Results and discussion

Data from 575 patients’ medical records were collected. Baseline demographic and clinical information of the study participants is summarised in Table 3. The study comprised mostly female patients (64%). The mean age (± standard deviation) for both men and women was 57 (± 11.38) years. The average baseline HbA1c for all participants was 8.78% (± 1.63). Mean baseline fasting plasma glucose concentration for the study population was 10.03 (± 3.62) mmol/l. The HbA1c findings of this study concur with those of another study done in three public sector academic teaching hospitals in Johannesburg, which investigated the management of type 2 diabetes mellitus and found a mean HbA1c of 8.7% for all study participants (Klisiewicz & Raal 2009). Similarly, the findings of a study performed in the private health care sector in South Africa (Amod et al. 2012b) reported an average HbA1c of 8.1% in participants with type 2 diabetes mellitus.

**TABLE 3:** Baseline demographic and clinical information of 575 type 2 diabetes mellitus patients receiving care at public health facilities in Cape Town.

Variable	*N*	%	Mean ± standard deviation	Minimum -maximum	Target range
Age (in years)	575	-	57 ± 11.38	29–92	-
**Gender**					
Male	206	36	-	-	-
Female	369	64	-	-	-
**HbA1C (%)**	493	-	8.78 ± 1.63	5.1–17.1	-
Low risk category (< 6.5)	13	-	8.15 ± 1.63	5.9–12	< 6.5%
Majority category (6.5–7.5)	219	-	8.80 ± 2.12	5.2–15.2	< 7%
High risk category (> 7.5)	261	-	8.75 ± 2.37	5.1–17.1	< 7.5%
Fasting plasma glucose (mmol/L)	570	-	10.03 ± 3.62	4.0–21.1	< 7 mmol/L
Systolic blood pressure (mmHg)	562	-	139.68 ± 21.82	101–217	< 140 mmHg
Diastolic blood pressure (mmHg)	562	-	79.91 ± 11.13	50–116	< 80 mmHg
Total cholesterol (mmol/L)	433	-	5.19 ± 1.25	1.1–9.4	< 4.5 mmol/L
**Body mass index (kg/m^2^)**	400	-	31.61 ± 6.16	17.93–56.36	< 25 kg/m^2^
Male	140	-	30.72 ± 5.71	19.14–50.27	< 25 kg/m^2^
Female	260	-	32.09 ± 6.35	17.93–56.36	< 25 kg/m^2^
**Comorbidities and complications**	575	-	-	-	-
Cardiovascular diseases	133	23	-	-	-
Ophthalmic diseases	2	0.3	-	-	-
Renal diseases	1	0.2	-	-	-
Peripheral vascular diseases	2	0.3	-	-	-
Complications (blindness)	1	0.2	-	-	-
T2DM only	42	7.3	-	-	-
Dyslipidaemia and T2DM only	17	3	-	-	-
Hypertension and T2DM only	377	65.6	-	-	-

T2DM, type 2 diabetes mellitus.

The mean systolic blood pressure of the study participants was 139.68 (± 21.82) mmHg and mean diastolic blood pressure was 79.91 (± 11.13) mmHg, which concurs with the latest targets of less than 140/80 mmHg in type 2 diabetes mellitus patients set out by the SEMDSA guidelines (Amod et al. 2012a). These findings concur with the findings of Amod et al. (2012b), who also found blood pressure was relatively well controlled, where participants (*n* = 701) with type 2 diabetes mellitus had mean systolic blood pressures of 132.9 (± 17.5) mmHg and mean diastolic pressure of 80 (± 10.2) mmHg (Amod et al. 2012b).

Table 3 also shows a mean body mass index (BMI) of 32.09 (± 6.35) kg/m^2^ and 30.72 (± 5.71) kg/m^2^ in women and men, respectively. This was expected as high BMI alone has been associated with type 2 diabetes mellitus and was also noted by Amod et al. (2012b) who recorded averages of 32.7 (± 6.8) kg/m^2^ for women and 31 (± 5.6) kg/m^2^ for men with type 2 diabetes mellitus in their study. These findings were higher than the BMIs reported in a community-based prevalence study on metabolic syndrome and diabetes mellitus done in Bellville South, Cape Town, during 2008 and 2009 on the general population, which found average BMIs of 30.8 kg/m^2^ in women and 25.8 kg/m^2^ in men (Erasmus et al. 2012).

The mean total cholesterol of the study participants (*n* = 433) was 5.19 (± 1.25) mmol/L, which is above the target of less than 4.5 mmol/L. In fact, 69% of participants (*n* = 300) had total cholesterol levels of above the 4.5 mmol/L target. Yet, only 3% of the participants were officially diagnosed with dyslipidaemia according to their medical records. This is in contrast to the study by Amod et al. (2012b), which found a prevalence rate of 62.5% for diagnosed dyslipidaemia of participants with type 2 diabetes mellitus. The under-diagnosis of dyslipidaemia in this study does not bode well for patients when considering that 59% of ischaemic heart disease and 29% of ischaemic strokes were attributed to high cholesterol levels in a burden of disease estimation study conducted in South Africa (Norman et al. 2007).

The most prevalent co-morbidities in this study were hypertension and cardiovascular diseases, with 65.6% and 23% of participants diagnosed, respectively. This is similar to the South African private sector study (Amod et al. 2012b), which also found hypertension to be the most common co-morbidity, with a prevalence rate of 77.6% of participants with type 2 diabetes mellitus. The prevalence of cardiovascular diseases in this study is of concern, because a systematic review by Girach and Vignati (2006) found that the presence of one microvascular complication usually predicts the development of another. The majority of T2DM patients in the study (93%) had been diagnosed with and were being treated for at least one other disease state. Only 7% (42) of the participants did not have other co-morbidities.

### Glycaemic control of participants

Table 4 shows that only 138 participants (28%) were within the HbA1c target ranges. Poor glycaemic control seems to be a trend in South Africa, with only 30% of patients in the private sector study (Amod et al. 2012b) and academic hospital study (Klisiewicz & Raal 2009) reaching a glycaemic target of 7%. There are a number of barriers to glycaemic control in patients with diabetes, including the occurrence and fear of hypoglycaemia and the complexity and demands of day-to-day management. These challenges have an enormous impact on patients’ quality of life, and health care costs are also considerable.

**TABLE 4:** Baseline glycaemic monitoring indicators of the study participants (*n* = 575) with glycated haemoglobin (HbA1c) results (*n* = 493) and fasting plasma glucose (FPG) levels (*n* = 574) stratified according to targets and allocated as per risk categories as set out for health facilities.

Categories	Stable		At risk	
	*n*	%	*n*	%
**HbA1c (%) (*n* = 493)**				
Low risk category (29–35 years) < 6.5%	2	0.41	11	2.23
Majority category (35–55 years) < 7%	42	8.52	177	35.90
High risk category (> 55 years) < 7.5%	94	19.07	167	33.87
**Fasting plasma glucose (mmol/L) (*n* = 574)**				
All categories	130	22.64	444	77.35

Similarly, fasting plasma glucose levels showed that only 23% of participants were normoglycaemic during their clinic visits. Nthangeni et al. (2002) also reported poor glycaemic control with more than half of their study participants having fasting plasma glucose above 8 mmol/L. This finding is again evident in the present study which was conducted more than 10 years later, indicating an urgent need for more effective glycaemic control.

During the study period of 18 months, the number of HbA1c tests performed per participant was not consistent throughout. HbA1c testing was done once in 493 participants, twice in 256 participants and three times in only 56 participants. Fasting plasma glucose was the predominantly used glycaemic indicator (574, 574 and 541), despite the limitation of FPG being able to show glucose levels for only few hours, whereas HbA1c measures glycaemic control over the preceding two to three months. According to the evidence-based guidelines, fasting plasma glucose should be measured at every visit. HbA1c should be measured six-monthly if the patient is at target or three-monthly if not at target, as well as whenever treatment is adjusted, and annually if the patient is stable. The clinical application of HbA1c in monitoring glycaemic control in patients with diabetes was already demonstrated in 1976 (Koenig et al. 1976). The measurement of HbA1c has become a standard in the care of patients with diabetes and for monitoring glycaemic control over a 3-month period. Aggressive improvement in glycaemic control, as demonstrated by a reduction in HbA1c, reduced the rate of diabetic complications and improved quality of life (WHO 2011).

**FIGURE 1:**
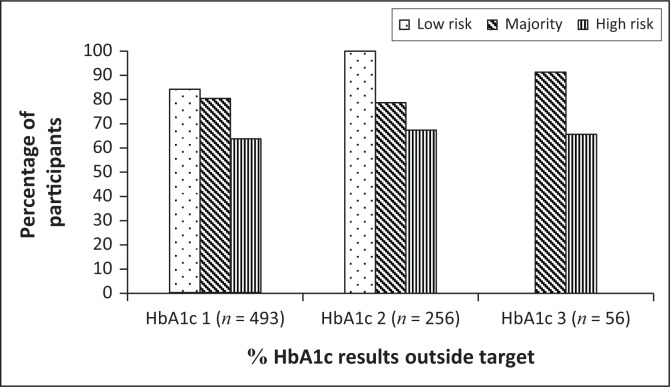
Percentage of participants with glycated haemoglobin (HbA1c) results outside target ranges during the 18 month follow-up period (*n* = 493 participants having one result, *n* = 256 participants having two results and *n* = 56 participants having three results).

Figure 1 shows the percentage of participants who did not meet the HbA1c targets over the 18 month follow-up period. These results showed that more than 60% of the participants over all the risk groups had HbA1c measurements that were consistently above the recommended targets. Similarly, fasting plasma glucose results showed no sequential differences that could have indicated an improvement or deterioration in diabetes management over the 18 month follow-up period. There was no statistical difference between the fasting plasma glucose means at three different intervals (using repeated measures ANOVA).

### Assessment of the pharmacotherapeutic management of participants

A total of three prescriptions per participant were recorded, which amounted to 1725 prescriptions for 575 participants. According to the number of the repeats of the three prescriptions (maximum of five repeats per prescription), the follow-up duration ranged from 12 to 18 months.

The percentage of participants that was on each regimen for prescriptions one, two and three were 39.3%, 37.57% and 38.96% on monotherapy, respectively; 41.04%, 42.96% and 40.87% on dual therapy, respectively; 10.09%, 9.91% and 10.78% on mono and insulin therapy, respectively; and 9.57%, 9.57% and 9.39% on dual and insulin therapy, respectively. None of the participants was on insulin mono therapy. This was not unexpected as patients on insulin mono therapy would probably not have been classified as stable and would require careful follow-up and monitoring. As in the private sector study (Amod et al. 2012b), metformin was the most commonly prescribed oral anti-diabetic agent with a range of 34.43%, 32.86% and 34.08% of participants (from prescriptions 1 to 3, respectively) on metformin alone in this study. In terms of patients on insulin, our study showed that about 20% of participants were on some oral combination and insulin compared to the private sector study, which found a prevalence of 34.53% of patients managed on insulin and oral agents.

Table 5 provides a summary of regimen changes recorded in all participants over the course of the three prescriptions. A total of 1150 potential regimen changes were available to be analysed during the follow-up period. In total, 72% of prescriptions remaining unchanged, 5.22% of prescriptions had a daily dosage increase and 1.48% a daily dosage decrease. Only 2.52% of prescriptions had step-ups in regimen and 2.7% step-downs in regimen, and 16.09% of prescriptions had lateral changes in their medication regimens.

**TABLE 5:** Treatment regimens and adjustments for three consecutive prescriptions in the 575 participants.

Treatment regimen	Prescription 1	Prescription 2	No change between P1 & P2	Daily dose increased	Daily dose decreased	Step-up regimen	Step-down regimen	Lateral regimen	Prescription 2	Prescription 3	No change between P2 & P3	Dose increased	Dose decreased	Step-up regimen	Step-down regimen	Lateral regimen change
**Mono therapy**																
Metformin	198	189	175	6	1	14	0	2	189	196	174	8	0	5	0	2
Gliclazide	23	20	17	1	0	3	0	2	20	13	11	0	0	1	0	8
Glimepiride	0	7	0	0	0	0	0	0	7	15	7	0	0	0	0	0
Glibenclamide	5	0	0	0	0	1	0	4	0	0	0	0	0	0	0	0
**Dual therapy**																
Metformin + Gliclazide	181	188	149	13	7	4	3	5	188	106	94	5	2	1	11	75
Metformin + glimepiride	11	30	8	0	0	0	2	1	30	115	26	1	0	0	1	2
Metformin + glibenclamide	44	29	22	1	0	0	1	20	29	14	13	0	0	0	3	13
**Mono + insulin**																
Metformin + insulin	51	50	32	7	2	0	0	10	50	56	39	2	1	0	2	6
Gliclazide + insulin	7	7	4	2	0	0	0	1	7	6	6	0	0	0	0	1
Glimepiride + insulin	0	0	0	0	0	0	0	0	0	0	0	0	0	0	0	0
Glibenclamide + insulin	0	0	0	0	0	0	0	0	0	0	0	0	0	0	0	0
**Dual + insulin**																
Metformin + Gliclazide + Insulin	43	42	22	6	3	0	3	9	42	26	13	6	1	0	4	18
Metformin + Glimepiride + insulin	4	8	3	1	0	0	0	0	8	26	7	0	0	0	0	1
Metformin + glibenclamide + insulin	8	5	4	1	0	0	1	2	5	2	2	0	0	0	0	3

**Total**	**575**	**575**	**436**	**38**	**13**	**22**	**10**	**56**	**575**	**575**	**392**	**22**	**4**	**7**	**21**	**129**

**FIGURE 2:**
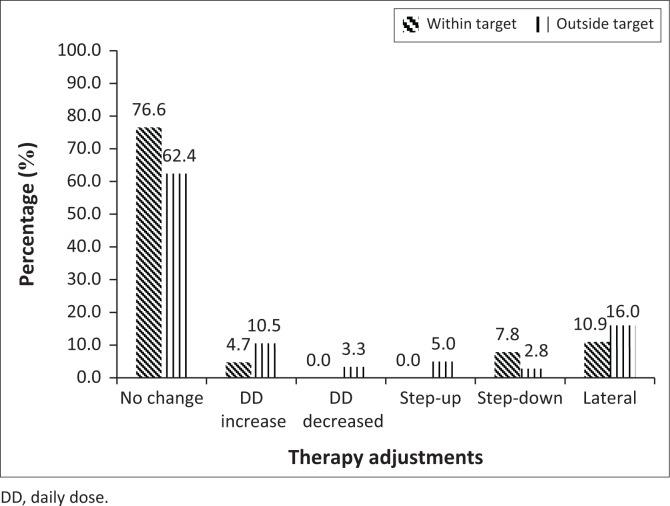
Summary of therapy adjustments in participants with glycated haemoglobin results which could have been correlated with the next prescription. Result totals represent 64 within target range and 181 results outside target.

Therapy adjustments were further classified according to the glycaemic monitoring indicator that was available at the time the prescription was written (e.g. if an HbA1c result was available at the time of writing the next prescription). In terms of HbA1c results that were available to the prescriber at the time of writing the next prescription, 245 results could be matched to 245 new prescriptions (Figure 2). Of these, 181 (70.7%) HbA1c results were outside target, and 64 results were inside target. Of the participant outside target, 15.5% had appropriate therapy adjustments, 78.4% had no change or a lateral change in their follow-up prescriptions, and 6.1% had therapy adjustments opposite to what guidelines suggest. For participants outside of target, more therapy adjustments, specifically dosage increases and step-up in regimen, would have been expected, while the opposite would have been expected for participants with results within target. For the 64 participants with results within target, no change is recommended if no side effects are experienced or if no medicine availability issues occur.

**FIGURE 3:**
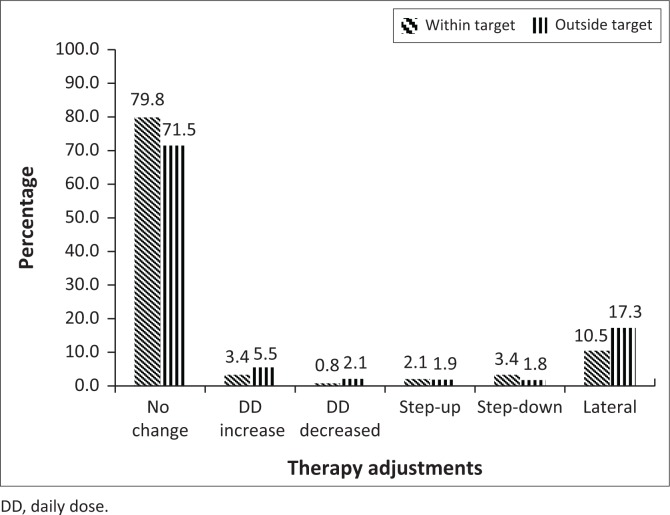
Summary of therapy adjustments in participants with fasting plasma glucose results which could have been correlated with the next prescription. Result totals represent 238 results within target and 852 results outside target.

Figure 3 shows the summary of therapy adjustments for the participants with fasting plasma glucose results for the 18 month follow-up period. Most participants had no changes in their prescriptions followed by lateral regimen changes. The total number of opportunities for intervention in participants with fasting plasma glucose results outside target was 852. The majority (71.5%) had not made therapy adjustments. There were 238 opportunities for interventions to happen in participants with fasting plasma glucose results within target; however, the majority (79.8%) noted no therapy adjustments.

This study did not find evidence to suggest that therapy adjustments were guided by glycaemic monitoring results of either HbA1c or fasting plasma glucose. In fact, 62.4% of participants with HbA1c levels outside the target range had no change in their follow-up prescriptions. This correlates with the results of a study in the United States that found that more than half of the patients with HbA1c results outside of target had no change in therapy (Wetzler & Snyder 2000). In accordance with Wetzler and Snyder’s (2000) comments, we also do not imply that good diabetes care is always equated to medication adjustments. Medication therapy adjustments need to be coordinated with patient participation and behavioural change (Wetzler & Snyder 2000), as well as a broad range of other interventions, which include improved access to health care, and promotion of a healthy lifestyle with the ultimate aim of reducing the burden of disease, increasing life expectancy, and improving the quality of life (Western Cape Government Health 2011).

### Limitations of the study

Due to the retrospective nature of the study, only the patient medical records were available for data collection which posed some limitations. These records were often incomplete. Date of first diagnosis was not available in the patient medical records; therefore, the duration of the participant’s experience of living with type 2 diabetes mellitus could not be established. Thus, co-morbidities or complications could not be linked to the duration. Additionally, information on lifestyle interventions such as adherence to medication, smoking, alcohol use, diet and physical activity was not collected, as the focus of the study was on therapeutic interventions. However, the aforementioned interventions are also very effective in improving glucose control.

## Conclusion and recommendations

This study investigated rational medicine use in the chronic management of 575 stable patients with type 2 diabetes mellitus at five different primary health care facilities in Cape Town. Fasting plasma glucose was the most used glycaemic monitoring indicator. Fasting plasma glucose monitoring provides information on glucose control for the preceding 8 h, and therefore cannot be used reliably to determine glycaemic control. In our study, patients had suboptimal glucose control. Glycaemic monitoring parameters were often not optimally used to guide medicine therapy adjustments. HbA1c not only provides a reliable measure of chronic hyperglycaemia but also correlates well with the risk of long-term diabetes complications. Elevated HbA1c has also been regarded as an independent risk factor for coronary heart disease and stroke in subjects with or without diabetes (Sherwani et al. 2016). Therefore, optimal use of HbA1c and fasting plasma glucose can prevent complications associated with type 2 diabetes. This could ultimately lessen the burden on the health system and economically benefit both the patient and health system, which is one of the goals of rational medicine use.

Health care workers should be educated on the appropriate use and interpretation of HbA1c at a clinic level, and exploration with prescribers of the barriers to following medication adjustment guidelines would be needed as well. Laboratory investigations recommended by treatment guidelines for the effective management of patients with diabetes, specifically those with type 2 diabetes mellitus, should be done accordingly. Adherence to treatment guidelines should be promoted to improve the rational use of medicines. Guideline implementation should be a priority for policymakers and facility managers. There are currently poor implementation practices seen at health facilities.
